# Exploring the contribution of lifestyle to the impact of education on the risk of cancer through Mendelian randomization analysis

**DOI:** 10.1038/s41598-024-54259-7

**Published:** 2024-03-13

**Authors:** Loukas Zagkos, Alexander Schwinges, Hasnat A. Amin, Terry Dovey, Fotios Drenos

**Affiliations:** 1 Department of Life Sciences, College of Health, Medicine and Life Sciences, Brunel University London, Kingston Lane, Uxbridge, London, UB8 3PH2 UK; 2 Department of Epidemiology and Biostatistics, School of Public Health, Imperial College London, St Mary’s Campus, London, W2 1PG UK; 3 Department of Infectious Diseases, Faculty of Medicine, National Heart & Lung Institute, Imperial College London, Cale Street, London, SW3 6LY UK

**Keywords:** Genome-wide association studies, Cancer genetics

## Abstract

Educational attainment (EA) has been linked to the risk of several types of cancer, despite having no expected direct biological connection. In this paper, we investigate the mediating role of alcohol consumption, smoking, vegetable consumption, fruit consumption and body mass index (BMI) in explaining the effect of EA on 7 cancer groupings. Large-scale genome wide association study (GWAS) results were used to construct the genetic instrument for EA and the lifestyle factors. We conducted GWAS in the UK Biobank sample in up to 335,024 individuals to obtain genetic association data for the cancer outcomes. Univariable and multivariable two-sample Mendelian randomization (MR) analyses and mediation analyses were then conducted to explore the causal effect and mediating proportions of these relations. MR mediation analysis revealed that reduced lifetime smoking index accounted for 81.7% (49.1% to 100%) of the protective effect of higher EA on lower respiratory cancer. Moreover, the effect of higher EA on lower respiratory cancer was mediated through vegetable consumption by 10.2% (4.4% to 15.9%). We found genetic evidence that the effect of EA on groups of cancer is due to behavioural changes in avoiding well established risk factors such as smoking and vegetable consuming.

## Introduction

Cancer is a risk to health and the primary cause of death worldwide^1,2^. An estimated 19.3 million new cancer cases and almost 10.0 million cancer deaths occurred in 2020^3^. A steady increase in mortality and incidence, particularly in developed countries^4^, calls for more effective cancer prevention^5^. While improvements in survival rates reflect progress in medical technology and healthcare, the rising incidence of cancer has been attributed to generational changes in obesity, lowered physical activity, a difference in diet and other lifestyle factors^6,7^. The fact that increasing cancer rates offset higher survival rates and lead to higher absolute mortality^8^ signifies the importance of tackling modifiable risk factors that lead to high incidence rates.

Educational attainment (EA) predicts cancer outcomes^9–11^ and is a central driver between socio-economic status (SES) and health^12^. For example, 22% of US cancer deaths could be prevented if all Americans had the cancer death rates of college-educated Americans^5^. Although there is no direct biological link between EA and cancer risk, it is believed that EA leads to more effective self-management and habits^12^. Various behaviourally related modifiable risk factors in low EA/SES have been studied. The most prominent factors identified include: alcohol consumption, physical inactivity, obesity, cigarette smoking, as well as low fruit and vegetable consumption^13–17^.

Studies specifically targeting the mediating effect of modifiable risk factors in EA and cancer risk are sparse. Some studies assess all-cause mortality^18,19^ while other studies address general cancer risk in the context of SES^20–22^. Quantitative assessments of the mediating effect of risk factors from such observational data are based on multivariable analysis, a method aiming to disentangle the effects of multiple variables on the outcome^23^. While the results of such assessments provide direction for further experimental studies, their validity is limited. Observational studies are also at risk of systemic biases between groups^24^. Furthermore, clustering of risky behaviours is common^25^, whereby individuals readily engage with a variety of behaviours, both risky and mitigating, the negative outcome that the authors wish to investigate, potentially introducing further bias. This makes it difficult to measure and correct for all risky behaviours and unmeasured risk factors might constitute confounders^26^ using traditional experimental investigative techniques. Moreover, data is commonly collected at distinct times, which does not capture a lifetime exposure^27^.

Mendelian Randomization is an analytical method to assess whether a risk factor has a causal effect on an outcome of interest, using genetic variants as instrumental variables^28^. The approach treats genetic variants as proxy measures for clinical interventions on risk factors, and thus has been extensively shown to anticipate the results of a randomised control trial^29^. The aim of this study was to explore the mediating role of lifestyle factors in explaining the effect of EA on the risk of cancer, using individual level data from the UK Biobank (UKB) and summary statistics from reliable published genome wide association studies (GWAS). We considered five lifestyle factors individually and simultaneously: number of alcoholic drinks per week, lifetime smoking index, BMI, fruit consumption and vegetable consumption. These five lifestyle factors were used to interrogate the underlying mechanisms by which EA affects the risk of cancer.

## Methods

### Data sources

#### Outcome data source

Individual level data on cancer incidence were obtained from UK Biobank (UKB), a prospective population study with detailed information about approximately 500,000 participants^30^. The data was collected between 2006 and 2011 and participants volunteered to provide biological samples for the measurement of biochemical markers and subsequent genotyping, anthropomorphic measures through a number of collection centres in the UK, and sociodemographic, lifestyle and health behaviours information through a series of in-person and online questionnaires. The processes for genotyping and data management have been described in depth^31^. Phenotype data were obtained from UKB data-fields 41,270 and 40,006 for 30 site-specific cancers. In this analysis, up to 335,024 UKB participants of European ancestry were considered, after excluding samples with relatedness of first or second degree and samples with discordant genetic and reported sex. Cancer cases were defined according to ICD-10 codes (international classification of diseases, 10th revision), obtained through linkage to national cancer registries. Genetic associations were estimated with 7 cancer groupings: digestive (7695 cases and 327,356 controls), female reproductive (3,612 cases and 177,190 controls), head and neck (1331 cases and 334,378 controls), lower gastrointestinal (GI) tract (6545 cases and 328,601 controls), lower respiratory (2307 cases and 332,457 controls), male reproductive (8988 cases and 149,131 controls) and upper gastrointestinal (GI) tract (1300 cases and 149,131 controls). Cancer groupings were generated to maximise statistical power and were determined based on their location in the body. Those with a cancer diagnosis were considered a case only for their chronologically first reported cancer type. This was done to distinguish between primary cancers originating in the specific tissue and secondary cancers metastasising from another location.

#### Exposure data source

To assess EA, we used publicly available summary statistics from a Social Science Genetic Association Consortium (SSGAC) meta-analysis of GWAS^32^. The primary meta-analysis combined 3 quality-controlled cohort-level results from studies in Europe and USA and was conducted on approximately 3 million individuals of European ancestry. Education years were measured for all samples over the age of 30. In this work, we used meta-analysis GWAS results from all discovery cohorts except 23andMe, conducted on approximately 800,000 samples, 442,183 of which were UKB participants.

#### Lifestyle data sources

Alcohol consumption, body mass index (BMI), smoking, fruit and vegetable consumption were assessed as potentially mediating risk factors in this work. Publicly available summary level data were used for alcoholic drinks consumed per week from Saunders et al.^33,34^, who conducted a GWAS meta-analysis using data from 60 cohorts on 2,965,643 individuals. To capture smoking behaviour, we used genetic summary statistics on lifetime smoking index, measured in 462,690 UKB participants of European ancestry who had phenotype data and passed genotype inclusion criteria^35^. Following a method previously reported^36^, smoking status, age at initiation in years, age at cessation in years and number of cigarettes smoked per day were combined into a lifetime smoking index. Genetic estimates for BMI were obtained from the Genetic Investigation of Anthropometric Traits consortium (GIANT) GWAS meta-analysis of 681,275 samples of European ancestry^37^, around 450,000 of which were UKB participants. To assess fruit and vegetable consumption, we used publicly available GWAS summary statistics from the MR-base^38^ for binary traits ‘fruit consumers’ and ‘vegetable consumers’, conducted on 64,949 UKB participants of European ancestry. These variables were generated as consumption over the last 24 h. A number of *Yes/No* questions relating to eating particular food groups or items were given to the participants following the pattern ‘*Did you eat any* < *food-group* > *yesterday?*’ providing examples of relevant foods and a picture. The participants completed the questionnaire in the assessment centre or online in four separate occasions within a year (http://biobank.ctsu.ox.ac.uk/crystal/docs/DietWebQ.pdf).

### Genome wide association studies

Genome wide association studies were conducted to obtain associations between 9,420,314 genotyped and imputed single nucleotide polymorphisms (SNPs) and 7 cancer groupings: digestive, female reproductive, head and neck, lower GI, lower respiratory, male reproductive and upper GI in up to 335,024 unrelated participants of white British ancestry. The SNPs tested were located in the autosomes. Only SNPs with a minor allele frequency greater than 0.01 and a Hardy–Weinberg equilibrium p-value greater than 10^–6^ were considered. The association of each SNP was tested using a linear regression model, adjusting for sex, age and the first 4 genetic principal components to control for population structure.

### Statistical analysis

#### Genetic instruments

Genetic variants were considered as instrumental variables for EA and the lifestyle factors in the MR analysis if they were bi-allelic, had a minor allele frequency (MAF) greater than 0.01 and a Hardy–Weinberg equilibrium P-value greater than 10^–6^. For EA, lifetime smoking index, drinks per week and BMI, genetic variants below the genome-wide significance threshold (p < 5 × 10^–8^) were selected as instruments, whereas for fruit and vegetable consumption, we considered a less stringent p-value threshold (p < 10^–5^), as it was the lowest threshold that provided robust signals for the two dietary GWAS results. We identified independent SNPs after clumping summary estimates, using a linkage disequilibrium (LD) threshold of r^2^ < 0.001 and a clumping window of 10 Mb. For the LD estimates between the genetic variants, we used the 1000 genomes phase 3 European reference panel^39^. To ensure that the first MR assumption holds, only genetic variants with an F-statistic greater than 10 were included in the analysis^40^. To further test the validity of the MR assumptions, we identified traits that associate with the genetic instruments used in this work, using the SNP nexus platform (https://www.snp-nexus.org/v4/). In the two-sample MR setting, genetic variants were excluded from the analysis when the direction of effects between exposure and outcome associations could not be inferred (in the case of palindromic SNPs with MAF greater than 0.42). Genetic instruments comprised 413 independent SNPs for EA, 507 independent variants for BMI, 126 for lifetime smoking index, 10 for drinks per week, 32 for fruit consuming and 21 for vegetable consuming.

#### Two-sample univariable Mendelian randomization

The current study used two-sample univariable and multivariable MR analysis in a multistep process to assess the relationships between EA, each of the 5 lifestyle factors and 7 cancer outcomes of interest. First, we tested the associations between EA and 7 cancer categories. We used categories over site-specific cancer types to maximise statistical power. Following this, we assessed the associations of EA with the 5 possible mediating lifestyle factors. Lifestyle factors were then tested against the 7 cancer categories simultaneously through MVMR. The random-effects inverse variance weighted (IVW) method was used as the main method for univariable MR analysis, which provides precise causal estimates, under the assumption that all genetic variants are valid instrumental variables^41^. The three instrumental variable assumptions dictate that the genetic instrument is associated with the exposure, is independent of any confounders of the exposure and outcome association and is associated with the outcome only via the exposure. In sensitivity analysis, we conducted the MR-Egger method^42^ to detect possible violations due to pleiotropic effects within genetic variants in the analysis and MR-weighted median method, which reports an accurate effect estimate, given that at least 50% of the weight in the analysis comes from valid instruments^43^. In addition, we performed MR-PRESSO, a method which detects and removes outlier SNPs based on their contribution to heterogeneity^44^. The I^2^ statistic was calculated to detect heterogeneity among the MR estimates obtained from multiple genetic variants. MR-IVW effect estimates were deemed statistically significant if association p-values were smaller than the Bonferroni corrected threshold 0.05/n, where n represents the total number of independent tests in each part of the analysis (EA with 7 cancer groups: *p* < 0.05/7 = 7.14 × 10^–3^, EA with 5 lifestyle factors: *p* < 0.01 and 5 lifestyle factors and EA with 7 cancer groups: *p* < 1.19 × 10^–3^). To quantify the amount of bias due to sample overlap in the MR effect estimates, we used the MRlap method^45^, which estimates corrected MR estimates, accounting for potential bias. We reported the p-value corresponding to the test statistic used to test for differences between the observed and corrected MR estimates.

#### Two-sample multivariable Mendelian randomization

To estimate the direct effect of each of the lifestyle factors on the risk of cancer, we performed multivariable Mendelian randomization (MVMR) analysis^46^. This method allows the use of multiple genetic variants associated with more than one risk factor as instruments to identify the causal effect of each risk factor on the outcome, independent of the rest of the risk factors. To obtain the list of genetic instruments for MVMR, we first merged, before clumping, the SNPs associated with each lifestyle factor or the exposure below their respective p-value thresholds as determined in the univariable MR analysis, and then clumped these genetic variants using for each variant the smallest p-value of their association with each lifestyle factor. Following this process, we generated a list of independent genetic variants that are associated with at least one lifestyle factor, as the MVMR paradigm dictates. All estimates were reported as odds ratio (OR) per unit increase in exposure, together with their 95% confidence interval (95% CI).

#### Proportion of lifestyle factor mediation

Network Mendelian randomization (network-MR) was conducted^47^ using the MR effect estimates and standard errors obtained previously to calculate the direct and indirect effect of EA on cancer risk. This was done per cancer outcome and per possible mediator, using their MVMR estimates, to obtain fractions of mediated and non-mediated effects. The indirect effect of EA on cancer through a lifestyle factor was estimated by multiplying the effect of EA on that factor times the effect of the lifestyle factor on the cancer outcome. The total effect was the estimated MR effect of EA on cancer. The direct effect was estimated by subtracting the indirect effect from the total effect, estimated from the first step MR. Last, the mediation proportion for each lifestyle factor was calculated by dividing the indirect effect over the total effect. To derive standard errors of the mediation proportion estimates, we used the delta method^48^.

#### Statistical software

Analysis was conducted in R version 4.0.2^49^, two-sample analyses and sensitivity analyses were performed using the “TwoSampleMR” v.0.5.6^50^ and “MRPRESSO” v1.0^44^ R packages. Figures were produced using the R package “forestplot” v3.1.1^51^. GWAS was conducted using PLINK 1.90 command line tool (www.cog-genomics.org/plink/1.9/). This study is reported based on the Strengthening the Reporting of Observational Studies in Epidemiology (STROBE) guidelines Supplementary Table 1.

### Ethics approval

This study is based on publicly available data and the informed consent and ethical review were acquired in all the original studies. The study is reported following the STROBE-MR statement.

## Results

**Figure 1:**
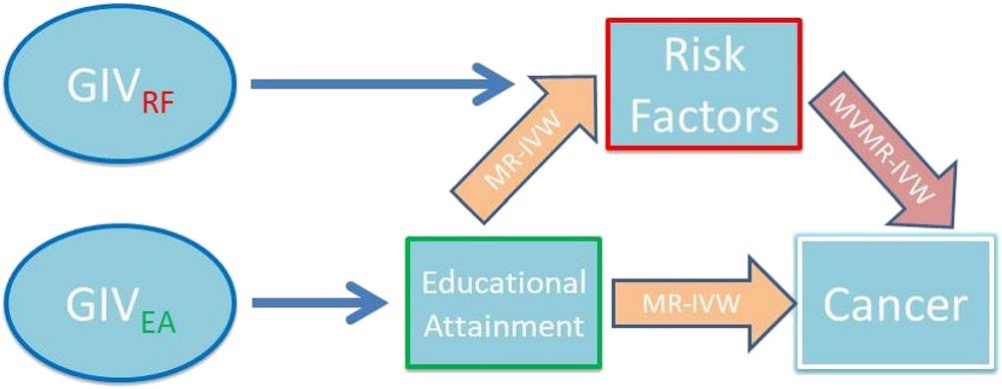
Study design. Network Mendelian randomization (MR) was conducted to identify the mediation proportion through lifestyle factors of the effect of education on the risk of cancer. Genetic instrumental variables were selected for each exposure based on their association below the genome-wide significance threshold, p < 5 × 10^–8^. To obtain robust signals, we used a less stringent threshold for fruit and vegetable consuming in the UKB (p < 10^–5^). Potentially causal estimates were produced using Mendelian randomization inverse variance weighted (MR-IVW) method as our main approach. MR sensitivity analyses were also conducted (MR-Egger, MR-weighted median, MR-PRESSO) to assess the robustness of the results. The simultaneous effects of each lifestyle factor on cancer were estimated using multivariable Mendelian randomization (MVMR-IVW). Mediation percentage through a lifestyle factor was obtained by dividing the indirect effect over the total effect.

Individual SNP estimates of the per-allele effects on EA, BMI, lifetime smoking index, drinks per week, fruit consumption and vegetable consumption are reported in Supplementary Table 2. Sample size, number of cases and included ICD-10 codes per cancer group are shown in Supplementary Table 3. Graphical representation of the model can be found in the directed acyclic graph (DAG) in Fig. 1. Univariable and multivariable MR estimates, sensitivity analysis results, MR-Egger intercepts and genetic heterogeneity statistics are provided in Supplementary Tables 4–6. Associations were considered statistically significant in this work if MR-IVW and MR-PRESSO estimates were significant after multiple testing correction, MR-Egger and MR-weighted median estimates had the same direction of effect and MR-Egger intercept was not significant (p > 0.05).

**Figure 2:**
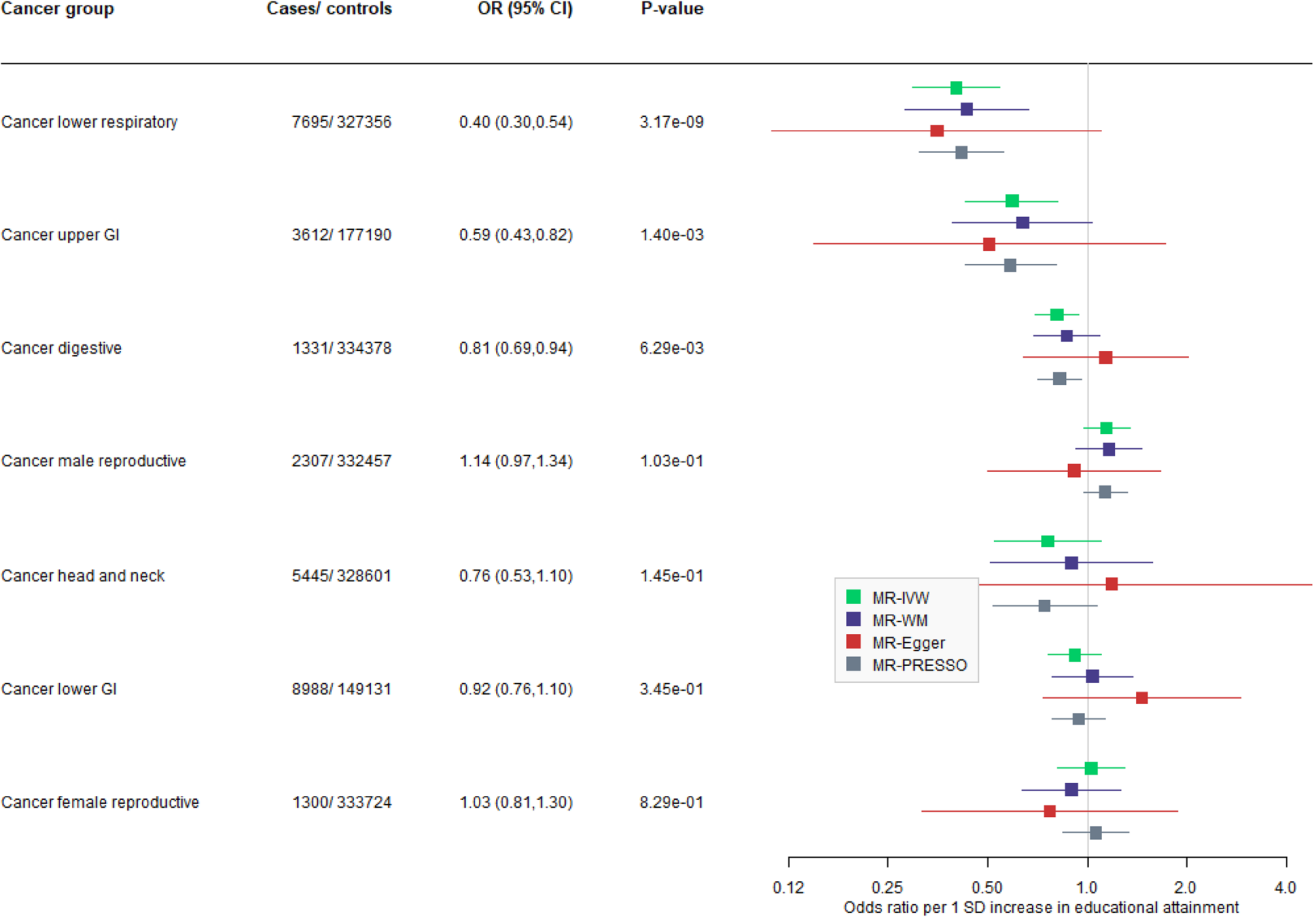
Two-sample Mendelian randomization (MR) estimates per 1 standard deviation increase in educational attainment for 7 cancer groups: digestive, female reproductive, head and neck, lower respiratory, lower gastrointestinal (GI) tract, male reproductive and upper GI tract. Associations were considered statistically significant if MR-IVW and MR-PRESSO p-values were smaller than 0.05/7 = 7.14 × 10^–3^, MR-Egger and MR-weighted median effect estimates were in the same direction and the MR-Egger intercept was not significant (p > 0.05).

**Figure 3:**
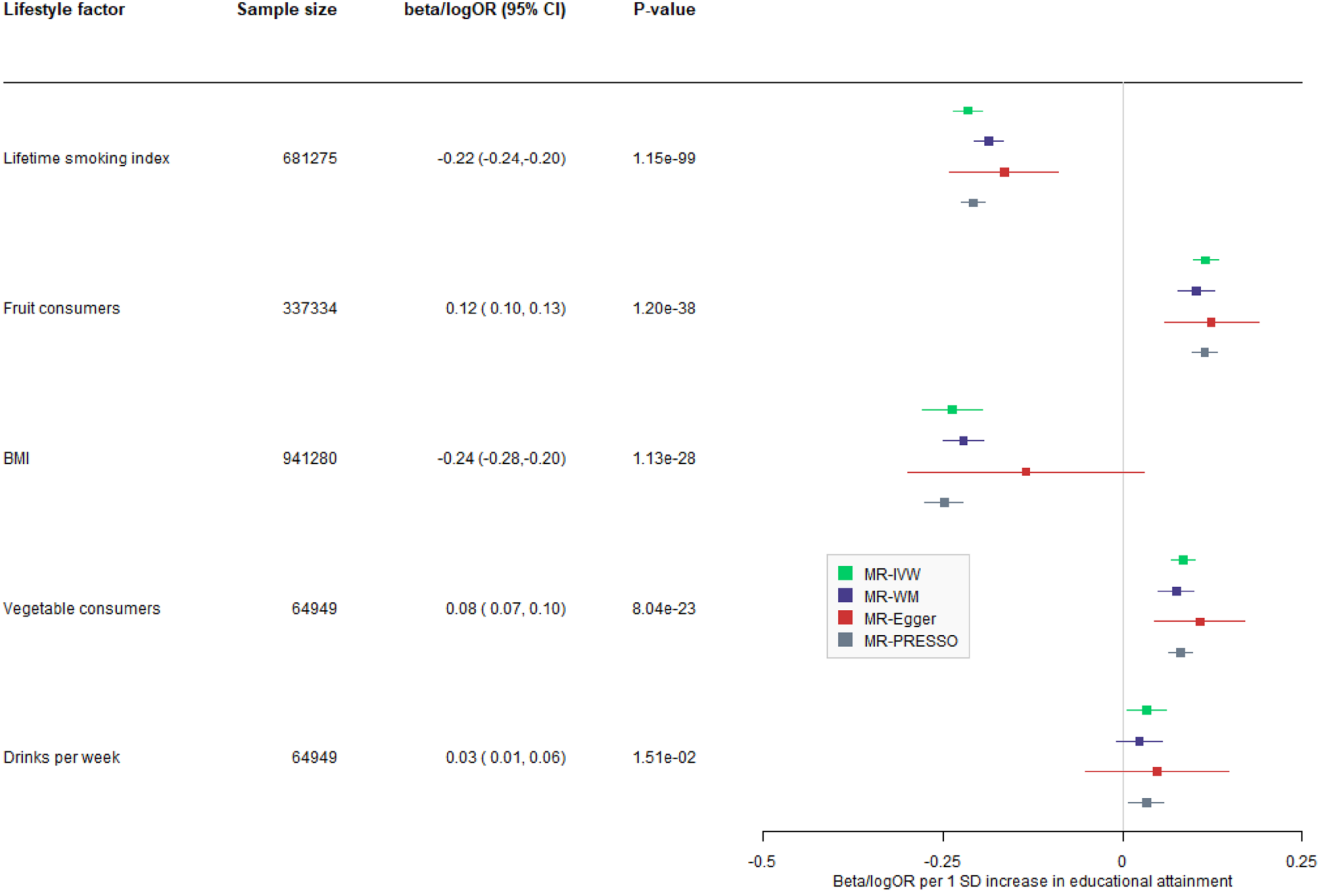
Two-sample Mendelian randomization (MR) estimates per 1 standard deviation increase in educational attainment for 5 lifestyle factors: body mass index (BMI), lifetime smoking index, drinks consumed per week, fruit consumed and vegetable consumed in the past 24 h. Associations were considered statistically significant if MR-IVW and MR-PRESSO p-values were smaller than 0.05/5 = 0.01, MR-Egger and MR-weighted median effect estimates were in the same direction and the MR-Egger intercept was not significant (p > 0.05).

The associations of EA with the lifestyle factors and the various groups of cancer were obtained per one standard deviation (sd) increase, which corresponds to 3.6 years of additional education in the UKB. MR-IVW results revealed two associations between EA and the odds of cancer, which were below the Bonferroni adjusted p-value threshold of p = 0.05/7 = 7.14 × 10^–3^. One sd increase in EA was associated with lower odds of lower respiratory tract cancer (OR: 0.40, 95% CI: 0.30 to 0.54), lower odds of upper GI cancer (OR: 0.59, 0.43 to 0.82) and lower odds of digestive cancer (OR: 0.81, 0.69 to 0.94) (Fig. 2). At a p = 0.05/5 = 0.01 Bonferroni threshold, MR-IVW results indicated that increasing EA was associated with lower BMI (beta: -0.24, -0.28 to -0.20) and lower lifetime smoking index (beta: -0.22, -0.24 to -0.20). One sd increase in EA was also associated with increased odds of fruit (OR: 1.12, 1.10 to 1.14) and vegetable consumption (OR: 1.08, 1.07 to 1.11). Weighted median and MR-Egger effects had similar effect estimates to the IVW method. The effect of EA on BMI was quite heterogeneous with an I^2^ statistic of 89% but a consistent effect between the MR-IVW, MR-Weighted median and MR-PRESSO methods. MR results of the effect of higher genetically predicted EA on lifestyle factors are summarised in Fig. 3. Moreover, using SNP nexus, the identified traits are unlikely to be potential confounders of the associations tested (Supplementary Table 7). MRlap method results suggested that there was no significant effect of sample overlap in the calculated MR estimates (Supplementary Table 8).

**Figure 4:**
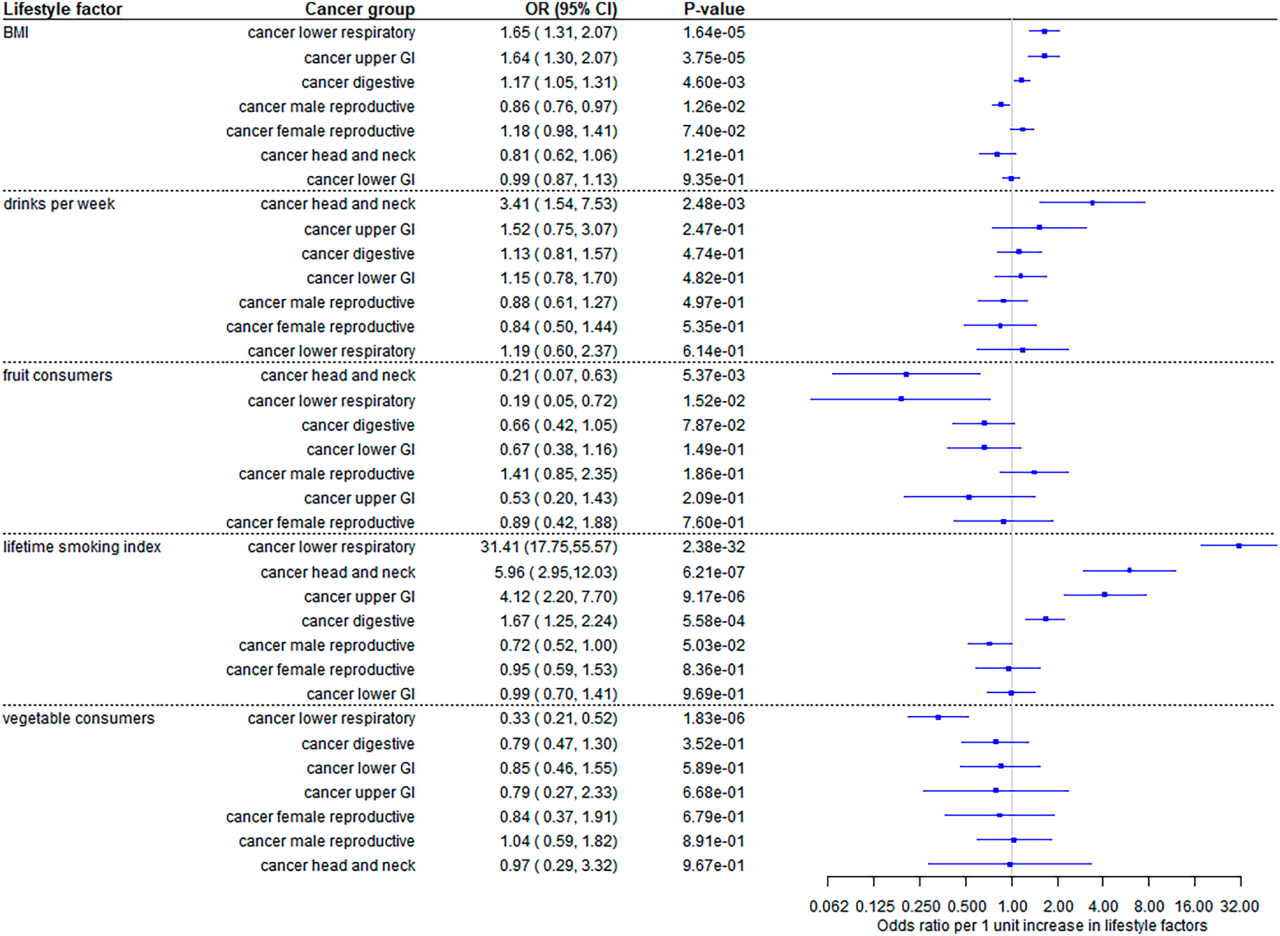
Two-sample multivariable Mendelian randomization (MVMR) estimates per 1 unit increase in 5 lifestyle factors for 7 cancer groups. Associations were considered statistically significant if MVMR-IVW p-values were smaller than 0.05/42 = 1.19 × 10^–3^.

MVMR analysis revealed seven significant effects of the lifestyle factors on the risk of cancer groups, below p = 0.05/42 = 1.19 × 10^–3^. Increasing lifetime smoking index was associated with increasing odds of lower respiratory cancer (OR: 31.4, 17.8 to 55.6), head and neck cancer (OR: 5.96, 2.95 to 12.03), upper gastrointestinal cancer (OR: 4.12, 2.20 to 7.70) and digestive cancer (OR: 1.67, 1.25 to 2.24). Higher genetically predicted BMI was associated with increased odds of upper GI cancer (OR: 1.64, 1.30 to 2.07) and lower respiratory cancer (OR:1.65, 1.31 to 2.07). Last, vegetable consuming was associated with lower odds of lower respiratory cancer (OR: 0.32, 0.21 to 0.52) (Fig. 4).

MR mediation analysis revealed two significant mediation ratios, after correcting for multiple testing (Supplementary Table 9). The largest part of the protective effect of increased EA on lower odds of lower respiratory cancer was mediated through smoking by 81.7% (50.5 to 100%). Interestingly, vegetable consumption was also mediating factor for the link between EA and lower respiratory cancer with 10.2% (4.4 to 15.9%).

## Discussion

The objective of this work was to identify the mediating lifestyle factors linking educational attainment to risk of cancer. We investigated 5 well supported lifestyle factors, including alcohol consumption, body mass index, fruit consumption, lifestyle smoking and vegetable consumption, using summary statistics from large cohorts. In our genetic analysis we found evidence of associations between EA and lower respiratory, upper GI and digestive cancers. MR mediation analysis results revealed that on average, 81.7% of the protective effect of EA on lower respiratory cancer was mediated by lifetime smoking and 10.2% by vegetable consumption.

The findings for the effect of EA on cancer risk are largely in agreement with literature. All potential causal associations found agree with estimates based on observational data found in literature^5,52–54^. MR methods therefore provide high-level evidence for a causal relationship between EA and cancer. Regarding the effect of EA on the lifestyle factors, the current study also supports existing findings. Previous comparisons of high-school and college EA estimated a 56%^55^ and 64%^56^ lower smoking status. Observational studies indicate that there is a negative association between EA and BMI in higher-income countries^57^. Existing literature provides no association estimates of EA with fruit and vegetable consumption but suggests an effect of socio-economic status on fruit and slightly lower on vegetable consumption^58,59^. Moreover, increasing EA is associated with increased alcohol intake frequency in MR studies^60^. The MR estimates of smoking on lower respiratory cancer are in agreement with observational studies^61^^,^^62,63^ for current smokers, but also with existing MR studies^64^. Moreover, genetically predicted BMI has been found to be positively associated with the risk of oesophageal cancer^65,66^. Previous observational study on mediation analysis for EA on lung cancer found that, adjustment for smoking decreased relative educational differences of lung cancer incidence by 50 to 70%^67^. Our MR network study gives a comparable mediation proportion of the protective effect of EA on lower respiratory cancer through lifetime exposure to smoking, around 80% of the total effect, since MR corrects for unknown confounding factors. Last, we identified a significant mediation proportion of the protective effect of EA on lung cancer through vegetable consumption, which is consistent with numerous observational studies suggesting a protective role of fruit and vegetables in lung cancer aetiology^68–70^.

Prior to making a number of key conclusions based on the data presented in this study, it is prudent to consider some of the limitations. The UKB sample has been shown to not be fully representative of the UK population. Individuals in the UKB have a higher likelihood, compared to the UK population, of being lean, non-smokers, non-drinkers and being older and female. This “healthy volunteer” bias is also affecting the total cancer incidence which may have introduced bias in our results. However, the assessment of effects of exposures on health outcomes in non-representative samples is still generalisable^71^. Fruit and vegetable consumption GWAS did not yield many strong genetic instruments compared to other factors possibly due to self-reported information and the limited time this accounts for. In addition, we excluded UKB participants from the lifestyle factors, where possible. However, partial sample overlap of EA, BMI, fruit and vegetable consumption with cancer incidence may have biased some of the MR effect estimates away from the null. Moreover, MR makes the assumption that all associations are linear, however, existing studies have shown a J-shape association between alcohol consumption and cancer risks^72,73^. In addition, the diagnosis of one cancer type may affect the surveillance, screening, or diagnostic practices for other cancer types. This could introduce bias if the ascertainment of the second cancer is influenced by the awareness or diagnosis of the first cancer. Last, due to the lack of availability of individual level data, we couldn’t test if there were any interactions between the exposure and the mediators.

The statistical power of a MR study depends on how much variation in the exposure is explained by the chosen genetic instrumental variables, the sample size and the true causal association between exposure and outcome^74^. Low cancer prevalence limits statistical power in this study, which may in turn explain the lack of statistically significant mediators identified beyond smoking and BMI. In addition, low vegetable and fruit consumption heritability could be limiting the usefulness of their genetic instruments. MVMR corrects for overlap in pathways, however, it is not able to deal with unmeasured confounders possibly interacting with associations^46^. Furthermore, MVMR could be subject to weak instrument bias^75^. A violation of the assumed linearity of causal effects could further bias the estimate^76^.

The results presented in this work provide several avenues of future research. The central limitation of statistical power has to be overcome with larger sample sizes or more accurate measurements. Further research on risk factors mediating cancer risk and EA as exposure also hinges on that requirement. Another potential, more methodological avenue of research is concerned with the addition of functional genetic variants to the developed pipeline. This would equip us with more tools to produce accurate estimates and validation thereof.

Medical science has rightly focused on how to treat people with cancer, however, any attempts to prevent the development of cancers are of utmost importance. Our finding, as well as work from others, indicate that the number of years in education has a proportional impact on cancer. Although it is not possible for everyone to reach the same level of EA, this work identifies our priorities in achieving similar benefits through targeted interventions. Currently, the detrimental effects of smoking and obesity are part of primary and secondary school curriculum in several countries. Given their relatively recent inclusion though, it is still early to quantify their effectiveness, as cancer is more common in older individuals that left education before the focus in healthier lifestyles. Nevertheless, our results suggest that we may see the gains of this strategy in the future and that a focus to educate children in primary and secondary education on the dangers of smoking and how to better maintain a healthy weight should be adopted more widely. In addition, investigations should tailor interventions to accommodate people that have already left education at secondary school level. Focusing our efforts of “good-health” education on the identified factors is likely to have the biggest impact on cancer rates.

### Supplementary Information

Supplementary Tables.

## Data Availability

UK Biobank individual level data used in this work can be accessed after applying for access at https://www.ukbiobank.ac.uk/enable-your-research/apply-for-access. Genetic association data are publicly available in the original studies.
